# Von der Funktion zur Versorgung: Der Barthel-Index als Annäherung an den Pflegegrad

**DOI:** 10.1055/a-2776-9797

**Published:** 2026-02-03

**Authors:** Maria Ivanova, Marie-Luise Rosenbusch, Juliane Neumann, Robert Arndt, Irmgard Landgraf, Doreen Müller

**Affiliations:** 1 Fachbereich Epidemiologie und Versorgungsatlas, Zentralinstitut für die kassenärztliche Versorgung, Berlin, Germany; 2 Fachbereich Versorgungsanalysen, Zentralinstitut für die kassenärztliche Versorgung, Berlin, Germany; 3 Zentrale Notaufnahme, Charité – Universitätsmedizin Berlin, Campus Benjamin Franklin, Berlin, Germany; 4 Hausarztpraxis Dres. Bajohr und Landgraf Berlin, Berlin, Germany

**Keywords:** Barthel-Index, Pflegebedürftigkeit, stationäre Leistungsdaten, Barthel index, need for care, inpatient service data

## Abstract

Basierend auf anonymisierten stationären Leistungsdaten aus dem Jahr 2023 aus dem InEK-DatenBrowser (gem. § 21 KHEntgG) wurde der Zusammenhang zwischen Einschränkungen der Selbsthilfefähigkeit – gemessen mit dem Barthel-Index – und dem zugewiesenen Pflegegrad untersucht. Ziel war es, zu prüfen, ob der Barthel-Index als praktikables Instrument zur Einschätzung der Pflegebedürftigkeit im ärztlichen Alltag genutzt werden kann. Die Ergebnisse zeigen, dass ein niedrigerer Barthel-Index signifikant mit einem höheren Pflegegrad assoziiert ist. Die ordinalen logistischen Regressionsanalysen (OR für Pflegegrad>1 zwischen 1,40 und 14,52) sowie der Kendall-Korrelationstest (τ=0,32; p<0,001; Cohen’s d=1,10) belegen einen statistisch signifikanten Zusammenhang. Damit kann der Barthel-Index als Indikator für die Einschätzung der Pflegebedürftigkeit herangezogen werden – insbesondere in Fällen, in denen noch kein Pflegegrad vorliegt. Diese Erkenntnisse können die ärztliche Entscheidungsfindung unterstützen und zur bedarfsgerechteren Versorgung beitragen. Zusätzlich könnten sie den ärztlichen Arbeitsalltag erleichtern, indem sie eine frühzeitige Identifikation einer Pflegebedürftigkeit ermöglichen.

## Abstract (en)

Based on anonymized inpatient service data from 2023 using the InEK-DatenBrowser (according to § 21 KHEntgG), the correlation between restrictions in the activities of daily living – measured with the Barthel Index – and the assigned level of care was examined. The aim was to demonstrate that the Barthel Index can be used as a practical tool for assessing the need for care in everyday medical practice. The results show that a lower Barthel Index is significantly associated with a higher level of care. Using ordinal logistic regression analyses (OR for level of care>1 are between 1.40 and 14.52) and the Kendall correlation test (τ=0.32; p<0.001; Cohen’s d=1.10) we were able to demonstrate a statistically significant correlation. Thus, the Barthel Index can be used as an indicator for care needs – especially in cases where no formal level of care has yet been assigned. These findings can support medical decision-making and contribute to more needs-based care. Additionally, they could potentially ease physicians’ daily routines by enabling the early identification of a need for long-term care.

## Einführung

In Deutschland können pflegebedürftige Personen Leistungen von Pflegekassen erhalten. Dafür ist eine offizielle Feststellung der Pflegebedürftigkeit erforderlich, die seit dem Inkrafttreten des zweiten Pflegestärkungsgesetzes im Jahr 2017 durch die Einteilung in fünf Pflegegrade erfolgt. Der Pflegegrad wird über den Pflegeaufwand ermittelt, der durch körperliche und kognitive Beeinträchtigungen im Bereich der Selbsthilfefähigkeit entsteht 1 .

Berichten aus der ärztlichen Arbeitspraxis zufolge besteht jedoch eine Hürde für Patientinnen und Patienten in Bezug auf Bürokratie- und Zeitaufwand, einen Pflegegrad zu beantragen. Das führt dazu, dass ihnen eigentlich zustehende Pflegeleistungen nicht abgerufen werden und Hilfestellungen nicht erfolgen können.

Im Alltag der ambulanten Versorgung ist die Behandlung pflegebedürftiger Personen ohne Pflegegrad erschwert, da auf bestimmte Pflegeleistungen – trotz tatsächlich vorhandener Notwendigkeit – kein von der Pflegekasse bewilligter Anspruch besteht. Insbesondere bei chronisch Erkrankten könnte der Zugang zu Pflegeleistungen zur Reduktion von Hospitalisierungen führen 2 .

Damit Ärztinnen und Ärzte im Praxisalltag schnell beurteilen können, ob die Beantragung eines Pflegegrads im jeweils vorliegenden Fall sinnvoll ist, bedarf es effizienter und unkomplizierter Methoden zur Einschätzung der Pflegebedürftigkeit. Der Barthel-Index (BI) – eine gewichtete Skala zur Messung der Funktionseinschränkungen im Alltag – kann zur Erfassung pflegerelevanter Behandlungsaufwände beziehungsweise der Pflegebedürftigkeit eingesetzt werden 3 .

Vor diesem Hintergrund stellt sich die Frage: Geht ein niedrigerer (schwerwiegenderer) BI mit einem höheren Pflegegrad einher?

## Methoden

Für die Beantwortung dieser Frage wurden anonymisierte Krankenhausdaten von 979 716 Fällen des Kalenderjahres 2023 aus dem InEK-DatenBrowser (gem. § 21 KHEntgG) verwendet. Dies umfasste Leistungsdaten zu allen abgeschlossenen voll- und teilstationären Behandlungsfällen bei Versorgung in Haupt- oder Belegabteilungen des Kalenderjahres 2023. Die ICD-10-GM-Diagnosen U50.00, U50.10, U50.20, U50.30, U50.40, U50.50 zur Dokumentation einer motorischen Funktionseinschränkung mittels BI wurden ausgewählt und allen Fällen mit den OPS-Codes 9–984 zur Bezeichnung des vorliegenden Pflegegrads gegenübergestellt (außer 9–984.b, da hier nur die Beantragung des Pflegegrads kodiert wurde). Dabei wurden pro Fall nur die schwerste vorliegende BI-Diagnose und der höchste vorliegende Pflegegrad berücksichtigt.

Zur Ermittlung des Einflusses einer schwerwiegenderen Funktionseinschränkung (niedrigerer BI) auf den Pflegegrad wurde eine ordinale logistische Regression eingesetzt. Zusätzlich wurde ein Kendall-Korrelationstest für ordinalskalierte Daten durchgeführt, um die Stärke des Zusammenhangs zu quantifizieren. Aufgrund der Beschaffenheit des InEK-DatenBrowsers kann die Stichprobe nicht anhand soziodemografischer Merkmale beschrieben werden.

## Ergebnisse

Die Ergebnisse der ordinalen logistischen Regression zeigen, dass ein niedriger BI signifikant mit einem höheren Pflegegrad assoziiert ist. Die Schwellenwerte für den Übergang in den nächsthöheren Pflegegrad ermöglichen eine differenzierte Trennung zwischen den Kategorien des Pflegegrads aufgrund des BI. Die Odds Ratios [und 95%-Konfidenzintervalle] dafür, einen Pflegegrad höher als 1 zu haben, sind für die verschiedenen Stufen des BI wie folgt: OR _80–95 Punkte=_ 1,40 [1,37; 1,43], OR _60–75 Punkte_ =2,15 [2,11; 2,19], OR _40–55 Punkte_ =2,98 [2,92; 3,03], OR _20–35 Punke_ =5,11 [5,01; 5,20] und OR _0–15 Punkte_ =14,52 [14,24;14,81].

**Abb. 1:**
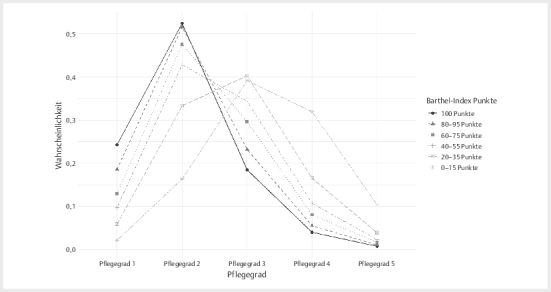
Es wird die Wahrscheinlichkeit abgebildet, für Fälle mit einem bestimmten Barthel-Index (100 Punkte bis unter 15 Punkte) einen bestimmten Pflegegrad (von 1 bis 5) zu haben. Die Wahrscheinlichkeiten für Pflegegrade pro Ausprägung des Barthel-Index summieren sich zu einer 1 auf. Die Punkte des Barthel-Index werden in 5er-Schritten aufsummiert; es können daher nur Werte, die durch 5 teilbar sind, erreicht werden. Interpretationsbeispiel bezüglich der Wahrscheinlichkeit verschiedener Pflegegrade: Bei einem Barthel-Index von 0–15 Punkten ist die Wahrscheinlichkeit am höchsten, den Pflegegrad 3 aufzuweisen (ca. 40%) und am niedrigsten, den Pflegegrad 1 aufzuweisen (ca. 2,5%).

Aus der Abb. 1 wird klar, dass in Fällen mit einem niedrigeren Pflegegrad auch weniger schwerwiegende Ausprägungen des BI wahrscheinlicher sind. Bei Personen mit einem BI zwischen 40 und 100 Punkten, die einen Pflegegrad aufweisen, ist der Pflegegrad 2 der wahrscheinlichste. Zwischen 0–40 Punkten ist bei Personen, die einen Pflegegrad aufweisen, der Pflegegrad 3 der wahrscheinlichste.

Das Kendallsche Tau bezieht sich auf die Korrelation von Rangreihenfolgen zweier Variablen in Bezug auf eine Population und kann als die Differenz der Wahrscheinlichkeiten, dass die Ränge zweier Variablen übereinstimmen (Konkordanz) beziehungsweise nicht übereinstimmen (Diskonkordanz), interpretiert werden 4 . Der hier erreichte Wert von 0,32 (p<0,001) zeigt, dass die Wahrscheinlichkeit eines Falls mit einem BI und einem Pflegegrad des gleichen Ranges (konkordantes Paar) höher ist als die Wahrscheinlichkeit, ein diskonkordantes Paar zu beobachten. Wenn man eine Rangfolge der Fälle über den BI bildet und anschließend die Rangfolge der gleichen Fälle über den Pflegegrad bildet, so ist die Wahrscheinlichkeit, dass die Fälle in beiden Variablen in einer ähnlichen Reihenfolge stehen, höher, als die Wahrscheinlichkeit, dass sie in einer unterschiedlichen Reihenfolge stehen. Je niedriger der BI ist, umso höher ist statistisch gesehen also der Pflegegrad.

Dieser Kendallsche Korrelationswert lässt sich in Cohen’s d transformieren, ein Wert der Effektstärke 5 . Effektstärken werden als Maß für inhaltliche und praktische Relevanz betrachtet 6 . Sie können aufgrund ihrer einfacheren Interpretierbarkeit die Einordnung eines Effekts erleichtern. Das Cohen’s d beträgt für die vorliegende Korrelation 1,10; ab Werten von über 0,80 werden Effektstärken als groß eingestuft 7 .

## Diskussion

Mit dem Vorliegen einer schwerwiegenderen BI-Diagnose erhöht sich die Wahrscheinlichkeit, einen höheren Pflegegrad aufzuweisen. Diese Ergebnisse unterstreichen die Bedeutung der funktionellen Fähigkeiten bei der Bestimmung des Pflegebedarfs und stimmen mit der Beobachtung überein, dass ein niedrigerer BI in der überwiegenden Zahl der Fälle zu einer Pflegegrad-Empfehlung führt 8 . Dies unterstreicht, dass der BI zur Einschätzung der Pflegebedürftigkeit genutzt werden kann, was auch international bereits gezeigt wurde 9 .

Obwohl der Zusammenhang zwischen BI und Pflegegrad mit einem Kendallschen Tau von 0,32 als moderat einzustufen ist, entspricht er einer Effektstärke von 1,10, die im Kontext der Versorgungsforschung als praktisch bedeutsam zu bewerten ist. In komplexen Versorgungssituationen wirken viele Faktoren in einem dynamischen und einflussnehmenden Kontext gleichzeitig. Ein Zusammenhang mit einer großen Effektstärke kann hierbei als entscheidungsrelevanter Befund gewertet werden.

Der BI ist bereits ein fester Bestandteil der ambulanten geriatrischen Versorgung, da er im hausärztlich-geriatrischen Assessment oft zur Beurteilung der Selbstversorgungsfähigkeiten genutzt wird. Da die hausärztliche geriatrische Betreuung nur bei regelmäßig durchgeführten Assessments abrechenbar ist, erscheint ein systematisches Screening zur Erfassung der Pflegebedürftigkeit bei älteren Patientinnen und Patienten notwendig. Die Verwendung des BI in diesem Kontext wird durch die vorliegenden Studienergebnisse gestützt und bietet eine ressourcenschonende Möglichkeit, frühzeitig Hinweise auf eine potenzielle Pflegebedürftigkeit zu erhalten und eine bedarfsorientierte Empfehlung zur Beantragung eines Pflegegrades auszusprechen. Dies kann sowohl im ambulanten als auch im stationären Setting – wo der BI bereits im Rahmen der neurologischen und geriatrischen Rehabilitation zur Einordnung in Rehabilitationsphasen häufig eingesetzt wird – erfolgen und trägt zur Sicherstellung einer bedarfsorientierten Versorgung bei.

Die schematische Anwendung des BI wird immer wieder kritisiert, da er in der aktuellen Form möglicherweise nicht für alle Patientengruppen, beispielsweise nicht für Demenzpatienten 10 , gut geeignet ist. Trotzdem wird er in vielen Kontexten angewendet und als praktikabel empfunden.

Mittelfristig könnte die Anwendung des BI in diesen Kontexten eine bedarfsgerechtere Ressourcenallokation fördern und die Versorgungsqualität in Anpassung an den tatsächlichen Versorgungsbedarf aufgrund funktioneller Einschränkungen der Patientinnen und Patienten verbessern.

## Ethikvotum

Die Nutzung der im InEK-Datenbrowser bereitgestellten Krankenhausabrechnungsdaten für wissenschaftliche Zwecke erfolgt auf Grundlage des § 21 KHEntgG. Ein Ethikvotum ist nicht erforderlich, da ausschließlich vollständig anonymisierte, aggregierte Routinedaten verwendet wurden, bei denen kein Personenbezug besteht.
